# Private in-patient psychiatry in the USA

**DOI:** 10.1192/pb.bp.113.044701

**Published:** 2014-10

**Authors:** L. Mark Russakoff

**Affiliations:** 1 Phelps Memorial Hospital Center, New York, USA

## Abstract

The US healthcare system is in the midst of major changes driven by four forces: the growing consensus in the country that the current system is financially unsustainable; managed care and parity legislation; the Affordable Care Act 2010; and the ageing of the ‘baby boomer’ generation. How these forces will combine and interact is unclear. The current state of in-patient psychiatric care and trends affecting the private practice of in-patient psychiatry over the next few years will be described.

## Background

Psychiatric in-patient care was historically provided at state and private hospitals by full-time psychiatrists at the facility. The lengths of stay in these hospitals were long - months, if not years. This pattern was dramatically changed in 1965 when federal monies became available through Medicare and Medicaid, which would pay for care in community hospitals, but not in institutions for patients with mental illness aged 21-64.^1^ At the same time, the modern era of psychopharmacology was born, permitting more effective treatment of the most serious disorders.^2^ The site of in-patient psychiatric care dramatically changed over the ensuing decades away from state hospitals to general hospital psychiatric units in both public and private hospitals. Nationwide, there has been a continued closure of state hospital beds with even more planned.^3^ For example, in 1955, New York State had an adult census of 93 197 in the state facilities. In 2003, the adult census was 4223.^4^ The number of private for-profit hospital beds grew, too.

Private in-patient psychiatric care in the USA now occurs primarily in two sites: specialty units in general community hospitals (often not for profit) and private psychiatric hospitals. In 2009, 3.1% of all persons aged 18 or older with any mental illness received in-patient psychiatric care, whereas 6.8% of those with serious mental illness were admitted to hospital.^3^ The rate of in-patient care of the general population has remained relatively stable between 0.7 and 1.0% from 2002 through 2009. This trend has occurred despite a decrease in the percentage of individuals 18 years and older who received any out-patient mental health treatment.

‘Nearly 60 percent of all mental health spending is from public funding sources’ (p.44).^3^ The percentage of mental health treatment expenditures for all in-patient care has decreased from 47.8% in 1986 to 29.1% in 2005, with a monotonic decrease over those 19 years, whereas total health expenditures have risen during that time frame.^3^ In many not-for-profit community hospitals which have in-patient psychiatric units, the majority of patients are covered by Medicare and Medicaid.

In 2012, it was estimated that healthcare costs in the USA accounted for 17% of the US gross domestic product, a rate that many feel is too high.^5^ The claim is made that much of these costs are avoidable waste in healthcare. A front page article in *The New York Times* lamented the huge cost of healthcare, estimated at $2.7 trillion,^5^ without concomitant excellent outcomes compared with lower-spending countries. In-patient care is a large contributor to these costs and efforts are being made to reduce ‘unnecessary’ in-patient stays. Readmission of acute myocardial infarction, congestive heart failure and pneumonia patients for any cause within 30 days of in index admission is penalised by the Centers for Medicare and Medicaid Services. These measures have not extended to psychiatric admissions as of this time. This penalty is being imposed despite the lack of evidence that these readmissions are truly preventable.

## Managed care, parity legislation and the process of care

Parity legislation (Paul Wellstone and Pete Domenici Mental Health Parity and Addiction Equity Act 2008) passed with the assumption that the mental health benefits would be managed. Health and Human Services Secretary Kathleen Sebelius announced on 8 November 2013 the final rules on how the parity legislation will be implemented under current healthcare reform.^6^ Thus far, many mental health organisations have endorsed these rules.

Many practitioners have felt that the management of mental health benefits has pushed the line from medical necessity to medically essential, that is, from the medically useful and desirable to the edge of malpractice. The criterion for admission to an in-patient service has become a determination of imminent dangerousness not clinical need *per se*. Admission to a psychiatric facility is viewed as a failure of the out-patient system, not a necessary and expectable component of a comprehensive treatment system for complex disorders.

These changes have affected the nature of in-patient psychiatric services and the processes of care.^7^ In the 1970s and 1980s, inspired by the work of Maxwell Jones, there was a focus on the in-patient unit as a community and on psychosocial interventions.^8^ It was common practice for patients to receive individual as well as group psychotherapy as part of their treatment. There was a spate of books published on how to organise and provide in-patient psychiatric care.^9-16^ More recent texts on in-patient psychiatry focus much less on the psychosocial and much more on the biomedical.^17,18^ Now, there is an almost exclusive focus on safety and rapid medication changes. Psychopharmacological interventions are accounting for a larger proportion of all psychiatric care. The number of prescriptions for antidepressants increased from 55.9 million in 1996 to 154.7 million in 2008. The corresponding increase in antipsychotics was from 9.3 million to 23.0 million.^3^

As soon as an in-patient is deemed not imminently dangerous, they must be discharged to a lower level of care. Psychosocial interventions now have more in common with crisis intervention and less with the treatment literature. With average lengths of stays hovering close to a week, the logistics of bringing in family members and arranging for aftercare have become the centrepiece of treatment. A model treatment plan for a length of stay of 5 days is proposed in a recent text of hospital psychiatry.^19^ From the viewpoint of third-party payers for hospital care (e.g. insurance companies), these changes wrought by managed care have been a success. Costs of mental healthcare are reduced. Mental health benefits can be offered on a par with non-mental health benefits without the fear of breaking the bank. From the perspective of families, the respite is unduly brief.

### Psychopharmacological interventions

However, psychopharmacological interventions require time to demonstrate efficacy. Antipsychotic medications require a week or more to establish their efficacy, although rapid early improvement is more the rule than the exception. Antidepressant medications require even more time to take effect. Current lengths of hospital stay are typically shorter than the times necessary to assess whether a medication or medication dose is effective. Short lengths of stay may rely on the non-specific effects of hospital care. After a brief time on medication, one can say that the dose appears to be tolerated. Because of the uncertainty of the specific effects of the medication, it makes timely and careful follow-up of discharged patients all the more important.

Clozapine changed the attitude of many psychiatrists towards polypharmacy - the prescription of more than one agent from a single class. It used to be uncommon for patients to be treated with more than one antipsychotic agent at a time. There were patients who had partially responded to clozapine monotherapy but were still symptomatic despite being at the upper limits of safe dosing of clozapine. Unable to safely increase the dose of clozapine, and desiring greater D_2_ receptor blockade, haloperidol was added to the regimen with many anecdotal reports of good responses. This particular combination became fairly common in the treatment of refractory patients.

Second-generation antipsychotics were modelled after what was thought to be the critical pharmacological characteristics of clozapine. When these new agents were used, and responses were not optimal, then, following what was ‘learned’ from the clozapine experience, combined second-generation antipsychotics began to be used with great frequency. If a non-sedating antipsychotic was initially used, and the patient appeared to be improving but anxiety or agitation persisted, a more sedating antipsychotic may be added, targeting those symptoms. Additionally, with greater sensitivity to possible mood components, antidepressant and mood-stabilising medications were often added. But these medications have been profoundly expensive. More and more psychotropics were released, each priced at a level which did not bring the overall prices down. Management of in-patient units required attention to the formulary as never before. The pharmaceutical companies took a primary role in educating psychiatrists, often over dinner, emphasising what was new and different, as well as what was problematic with previously available agents, and many psychiatrists were early adopters of new medications. Many specialty psychiatric units are now reimbursed for psychiatric care on a *per diem* basis. The cost of medications is not a pass-through expense. The pharmacy expenses exploded, eating into whatever profits were found from the in-patient psychiatric services. With the release of generic atypical antipsychotics, costs have dramatically dropped, but the damage to the bottom line had already been done.

### Rapidly processing patients

The pressure to rapidly process patients through a hospitalisation has forced rapid medication decisions. Whereas in years past, a patient was often admitted and then observed for a couple of days without any specific medication treatment in order to clarify the diagnosis, this practice was seen as wasteful. Since a large portion of persons admitted to psychiatric units in acutely psychotic states are also substance misusers, patients who are admitted because of exacerbations of psychosis caused by substances are nevertheless often given even more medication as if their ongoing dosage was inadequate. These sequences have contributed to the polypharmacy described above. Since many of the newer drugs of misuse - bath salts, for instance - are not identified in routine urine toxicology tests, and patients often do not know what substances they have consumed, the causes of the exacerbations are often never understood. But the treatment is often more antipsychotics.

### Discharge planning

Discharge planning is hampered by the need to develop and effect a plan with very little time. Unfortunately, discharge planning is sometimes closer to discharge theory - a plan that makes sense but cannot be implemented because of time pressures on providers, families and the aftercare agencies. Often there are barriers to access-needed care because of insurance problems - the person is uninsured, the lists of participating providers for the patient’s insurance are inaccurate, and the patient does not have the documentation to complete their application for out-patient Medicaid - and placement. Access to housing is a problem for the mentally well; for the mentally ill, it is a nightmare. There is a dearth of housing available to people, especially those on limited income. In metropolitan areas that are expensive, the pressures are enormous. Shelter systems are in place but the nature of the placement is such that the experience is not conducive to stability and continuity of care. Housing First programmes - in which a mentally ill person is placed in housing with few contingencies - have been reported to reduce in-patient utilisation, but funding is limited.

### Staff motivation

With patients at higher acuity, and staff attention diverted to managing the managed care reviewers, less time is available to treat the patients. Unit leadership is challenged to keep staff motivated and not to burn out. The value of the staff’s efforts needs to be reinforced. Since the staff are less likely to see the fruition of their efforts - to see patients become well - the focus needs to be on the value of the elements of care, including their role in the value chain and dealing with managed care companies, to combat their sense of frustration.

## Fiscal pressures and lack of psychiatric beds

Hospital facilities have been subject to unrelenting forces to decrease their costs, with reductions in reimbursement and demands for frequent reviews of the medical necessity of continued stay. If an in-patient unit cannot be filled, either because of greater restrictions on admissions, reduced lengths of stay or more effective out-patient services, then the unit’s viability is jeopardised. In 1997, there were 1478 community hospitals with mental health/substance misuse units. By 2006, there were only 1201; preliminary data for 2007 indicated a further decline to 1180.^3^ The number of general hospital psychiatric beds per 100 000 civilian population dropped from 21.9 in 1990 to 13.9 in 2004; the corresponding number of private psychiatric beds per 100 000 civilian population dropped from 18.4 to 9.5. The number of private psychiatric hospitals peaked in 1992 at 475. By 2004, the number was reduced to 264.^3^ In 2000, in Westchester County New York, a relatively affluent county just north of New York City, there were four private for-profit psychiatric hospitals; today, there is only one. In 2013, a private for-profit psychiatric hospital in New York City closed its doors when it failed to successfully negotiate a new contract with its largest union.

In the past, a well-run psychiatric in-patient unit could be a modest profit centre. The landscape has changed dramatically for general hospitals. The chief financial officer will tell you that the psychiatric units do not perform as well as the medical surgical units. The psychiatric unit occupies space where more lucrative medical surgical services, such as invasive cardiology and advanced surgery patients, could be located. A number of hospitals have made the business decision to close their psychiatric units in favour of these more remunerative services. These decisions are not simply evidence of greed. The profit margins of most general hospitals are razor thin, well below what Wall Street would accept for a company. Hospitals need to generate money where they can, and that includes consideration of the opportunity costs of low-performing units.

Many psychiatrists complain of the lack of psychiatric beds. The closing of state, county and general hospital psychiatric units has contributed to the problem of psychiatric patients being held - boarded - in emergency rooms for days on end. The situation of bed availability varies dramatically around the USA. Most emergency rooms are not built to safely hold psychiatric patients for days; nor are these environments conducive to quick recovery. Patients who present in intoxicated, agitated states may be safely managed in such situations until they become sober, but not those psychiatric patients who present in a sober but agitated and psychotic state. There has been a transinstitutionalisation of patients from public in-patient facilities to jails and prisons, particularly of those patients who become victims of the ‘revolving door’ of psychiatric admissions, rapid treatment and discharge, and then readmission when they do not continue in out-patient treatment and take their medications. The loss of in-patient capacity has not been matched by substantially enhanced out-patient services geared to keeping the patients in the community.

There has also been a loss of private not-for-profit beds. Much of this loss was the effect of managed care and changing standards of in-patient care. Facilities that specialised in long-term in-patient care - many not for profit - were especially hard hit. Many of these hospitals simply closed their doors. Of the surviving institutions, some have re-invented themselves in a highly creative way, not giving up their broader mission but meeting their goals through other means. Some have converted hospital beds into residences, so the patient becomes an out-patient while residing in what was a hospital bed, which may be paid for as if it were a hotel. Others have yielded to the pressures of managed care, dramatically shortening their length of stay and marketing themselves to a broader sector of the population so that they can keep their beds filled and their institutions viable. One institution moved to another state and became affiliated with a medical school.

## Staffing issues

In the past, many psychiatrists would care for their private patients when they needed hospital admission. They maintained privileges at a local hospital, often serving on call as part of their professional responsibilities. Early on, managed care required doctors to have hospital privileges, adding to the cadre of psychiatrists on call. When the criteria for admission were more related to clinical necessity and degree of psychic pain, being on call could be a source of referrals as well as a community service. When the criteria for admission became tied to imminent dangerousness, the population of patients seen in the emergency room and being admitted became less desirable and suitable for solo practitioners. The requirement for having hospital privileges was dropped by many managed care companies. As reimbursement for therapy sessions was reduced, it became less cost-effective to take time away from one’s out-patient practice to tend to patients who had been admitted. Thus, many private psychiatrists no longer have clinical privileges at any hospital. The loss of these psychiatrists has made psychiatric cover of emergency rooms thinner. Non-psychiatrists often provide cover with telephone consultation from a psychiatrist if requested. Facilities that can afford to do so hire ‘moonlighters’. (In psychiatry, moonlighters may be advanced psychiatric residents or psychiatrists with primary incomes derived from other sources.) Telepsychiatry is in its infancy and likely to play a larger role in the future.

There have also been changes in the culture in all of medicine regarding on call. Resident hours were reduced because of the concern that prolonged service periods - often 36 h - contributed to serious medical errors and poor learning experiences. Residents are now trained with the idea that one does not attend one’s patients continually, and that there are frequent hand-offs of patients to other residents. Continuity by a single provider is no longer a revered principle of care but seen as an anachronism predicated upon fictions of ‘super’ doctors of yesteryear. A typical scenario is for a patient to be admitted after hours by an on-call psychiatrist, often a moonlighter. The next day, the patient is assigned a psychiatrist. If the patient stays in the hospital for a week, then a different psychiatrist - or two - sees the patient over the weekend. Thus, for a patient who is in hospital for a week, up to four psychiatrists could have seen the patient! Nurses may work three 12-hour shifts per week; continuity of care is primarily continuity of the institution and not of the providers. The personal relationship with the psychiatrist and the nurse has been thus diluted.

However, the convergence of the elimination of the need for hospital privileges, the change in the population seen in the emergency room and on in-patient services, the reduced reimbursement for sessions and the cultural changes, contributed to many private psychiatrists severing their affiliations with hospitals and letting their patients be cared for as in-patients by psychiatrist hospitalists (hospitalists are doctors who solely treat in-patients; they are hospital care specialists). These changes institutionalised fragmentation of care of all, regardless of ability to pay.

Current hospitalists have become adept at dealing with managed care, rapidly medicating patients to mitigate the most serious and prominent symptoms and facilitating the discharge of patients to lower levels of care. Since the time frame for all this to occur is brief, and since psychiatrists often do not answer telephones during consultation sessions, there is inadequate time for there to be reasonable communication among providers regarding treatment plans. This schism between in-patient and out-patient care persists, unmitigated by the use of electronic medical records. The move to a model in which in-patient treatment is provided by hospitalists has extended to general medicine, too, with parallel problems in continuity of care.

## Healthcare reform - the Affordable Care Act 2010

Will the Affordable Care Act 2010 change the nature of in-patient psychiatric practice? The initial legal challenge to the Act was rejected by the US Supreme Court in June 2012, but other challenges are wending their way through the courts. The Act has already led to more young adults having private insurance through their working parents, which may change some of the earlier quoted numbers. The promise of increased access through increased numbers of insured patients via the expansion of Medicaid will not be delivered across the country as a number of states have rejected the expansion. However, for those states who have embraced the expansion, the market may expand suddenly, especially in areas where there have been many eligible and uninsured individuals. It is unclear whether providers will be able to accommodate large increases in patients should that occur. On the other hand, the roll out of the federal health insurance exchange has been fraught with problems, which are currently being corrected. Whether the pushback from those problems, combined with the cancellation of policies which do not meet the Act’s standards for insurance, may yet lead to unpredictable changes in the course of reform.

The Affordable Care Act pressures providers to organise care organisations and provide health homes and integrated care systems for comprehensive care of patients. The health homes are expected to provide care management for all patients. It is unclear how mental health treatment, including in-patient treatment, will be managed in these structures. There are very few mental health systems of care that are integrated at this time. Many in-patient units accept patients from a broad geographical area. Traditional psychiatric case management services have been helpful in assuring that recently discharged patients receive follow-up care. The staffing ratios for these case managers are much higher than what is proposed for health homes and these case managers have expertise in dealing with psychiatric patients and often visit patients. Already, behavioural health managed care companies are partnering with risk-bearing Act entities. Medicaid for the chronically mentally ill will be subject to managed care, even though very few people have any experience in doing so.

Although one of the requirements of healthcare reform in the USA was to have interoperable electronic medical records, this goal has been a fantasy and not a fact. Most psychiatrists in private practice do not have electronic medical records. The records of a private psychiatrist are rarely available when a patient presents in the emergency room. Even if it is possible to reach the psychiatrist out of hours, the psychiatrist may not have available the latest information regarding the patient’s medication regimen.

If the patient is seen in the same institution in which they receive their psychiatric care, the electronic medical records are often a product of serial ‘cut and paste’ histories, which are often incoherent, wrong and do not provide the information needed in the emergency room to make a proper decision. The excessive use of cut and paste, cloning of notes and failure to create an integrated statement of the patient’s problems is not unique to psychiatric record keeping. Although the US federal government has promoted electronic medical records by paying adopters to meet ‘meaningful use’ criteria, these criteria are easily met without the records being clinically meaningful. It becomes one of the great ironies that monies are awarded for ‘meaningful’ use when they are anything but that. With the use of extensive templates and checklists, psychiatric records generated by electronic systems are now quite detailed, sterile, read poorly and often devoid of critical information for the next practitioner. Rather than rendering communication more efficient, the records now obfuscate the transmission of the important information. Because of this, they are even less likely to be read by a busy clinician.

Regulations that have an impact on in-patient care are unrelenting. The Affordable Care Act promotes a focus on population health. It remains to be seen how it will directly affect in-patient care beyond what has already been said. The Substance Abuse and Mental Health Services Administration promotes the recovery model which requires individualised treatment planning, patient-centred care and empowering patients. But there is disconnect between these principles of recovery and the principles of efficiency and evidenced-based care. In-patients are not necessarily inclined to request evidenced-based treatments, nor feel comfortable with the time frames imposed by managed care. For many patients, compliance is a challenge. For those who are admitted because of a lack of adherence to their medication treatment, one does not instil adherence within a few hours. Motivational interviewing techniques are helpful but not rapidly effective in leading to attitudinal change. Not infrequently, the act of admission mitigates some of the severe symptomatology which may diminish the experienced pressure to take the recommended medications. In-patient care had traditionally been a site for asylum and respite, but with lengths of stays reduced to about a week, unit milieus chaotic, and with the decision to discharge being debated with managed care almost daily, there is no time to relax.

Electronic records can facilitate standard documentation but if the requirement is to write treatment goals in the patient’s words, there are no efficiencies from the electronic record. The clinician who is not a skilled typist suffers in productivity. Typing skills and ease of use of a computer are as basic to modern in-patient psychiatric practice as is an understanding of pharmacology. An extraordinary amount of time is now needed to complete required documentation. Electronic records have too often not improved efficiency or productivity. If one uses concomitant documentation - writing the note during the session - and is not a touch typist, then the doctor’s eye contact is poor, and some patients feel that the doctor does not care; spontaneity is lost.

## Ageing of the ‘baby boomers’

The ‘silver tsunami’ - ageing of the baby boomer generation - will create new pressures on emergency rooms and in-patient psychiatric services.^20^ Geriatric patients typically have more medical comorbidities than younger adults. Psychiatric services need to be physically prepared to house more patients with physical disabilities (e.g. more bathrooms and showers for disabled people). Similarly, units need to be staffed to accommodate these patients’ needs (i.e. more patient care aides to assist with dressing, toileting, etc.). Access to general medical care and laboratory testing may need to be enhanced as well.

If a patient has schizophrenia, bipolar disorder or depression and happens to have dementia, and those symptoms from their non-dementia psychiatric illness warrant admission, then those patients may be admitted to a psychiatric unit for treatment. However, if the primary diagnosis is dementia, and the symptoms that are driving the request for admission stem from the dementia, then many state regulations indicate that those patients are not to be admitted to a psychiatric unit. Patients whose primary diagnosis is dementia are not to be admitted to psychiatric services, as such patients are expected to be treated in nursing homes.

However, many nursing homes are neither designed nor staffed to manage such patients and these patients are regularly sent from nursing homes after an incident to the emergency room with the hope that they will be admitted. Emergency room staff and consulting psychiatrists are placed at odds with one another. Internal medicine staff do not want to admit these patients to the general medical service for ‘altered mental status’ because such patients require fairly intense supervision, for which medical units are not staffed. While the debate goes on, the patient is often sleeping in the emergency room or asking to go home. State regulators - who implement the restrictions on admission - state that nursing homes are paid to care for such patients, and look askance at any admission to a psychiatric bed from a nursing home, feeling that effective care could be delivered in the nursing home. However, many nursing homes have very limited hours of psychiatric consultation time and psychiatrists are not willing to manage a patient who needs daily assessments. These problems, pressures and conflicts are likely to grow over time.

## Conclusions

In summary, private in-patient psychiatric care is continuing to undergo many changes, and more are likely to come. To function within the fiscal constraints, more physician extenders (e.g. nurse practitioners, physician assistants) are likely to be hired and perform functions previously performed by psychiatrists. The shortage of psychiatric beds is likely to worsen, which will pressure new systems of care into creating more cost-effective interventions. The movement from the in-patient service being a site of biopsychosocial comprehensive care will continue to narrow to intensive pharmacological interventions and electroconvulsive therapy. If the Affordable Care Act delivers its promise of integrated, coordinated, patient-centred continuous care, then patients will benefit enormously. It remains to be seen whether resources can be reallocated efficiently and effectively without unacceptable amounts of collateral damage.
